# Management of rhinophyma

**DOI:** 10.1093/jscr/rjaf280

**Published:** 2025-05-09

**Authors:** Sarra Benwadih, Maha Bouksirat, Bouchra Dani, Malik Boulaadas

**Affiliations:** Maxillofacial Surgery Department, Hospital of Specialties, Mohammed V University, Rabat 10100, Morocco; Maxillofacial Surgery Department, Hospital of Specialties, Mohammed V University, Rabat 10100, Morocco; Maxillofacial Surgery Department, Hospital of Specialties, Mohammed V University, Rabat 10100, Morocco; Maxillofacial Surgery Department, Hospital of Specialties, Mohammed V University, Rabat 10100, Morocco

**Keywords:** rhinophyma, rosacea, skin thickening, nasal deformity, sebaceous glands, electrosurgery

## Abstract

Rhinophyma is a chronic skin condition that predominantly affects the nose, characterized by thickening and fibrosis of the skin, leading to a bulbous, enlarged appearance. It is typically associated with rosacea, though its exact etiology remains unclear. Our department recently managed two cases of rhinophyma, which allowed us to explore the clinical presentation, diagnostic challenges, and treatment strategies. This report summarizes the key aspects of these cases and offers insights into the management of rhinophyma.

## Introduction

Rhinophyma, derived from the Greek words “rhis” (nose) and “phyma” (growth), refers to the enlarged, bulbous [1], and reddened appearance of the nose, typically resulting from granulomatous infiltration, often as a consequence of untreated rosacea. Rhinophyma is relatively rare typically affects individuals in their 40s to 50s and is relatively uncommon before the age of 30 and It is most frequently seen in Caucasian populations, particularly individuals of Northern European descent.

The commencement is subtle and the progression is chronic [2]. The fundamental problem the patients present with is cosmetic deformity. Clinical evolution is slow and often neglected by patients. In the most severe cases, in addition to the esthetic prejudice and its social repercussions, patients consult us for functional reasons, starting with nasal obstruction [3] in addition to breathing and vision difficulty. Our aim, based on this case, was to review the physiopathology, clinic and treatment of this pathology.

## Case presentation

**Figure 1:**
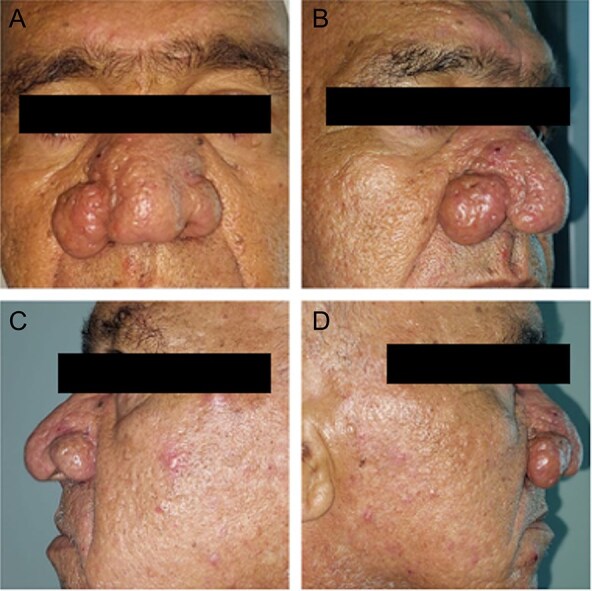
Patient 1. (A) Patient front before the surgery. (B, D) Patient right profile before the surgery. (C) Patient left profile before the surgery.

**Figure 2:**
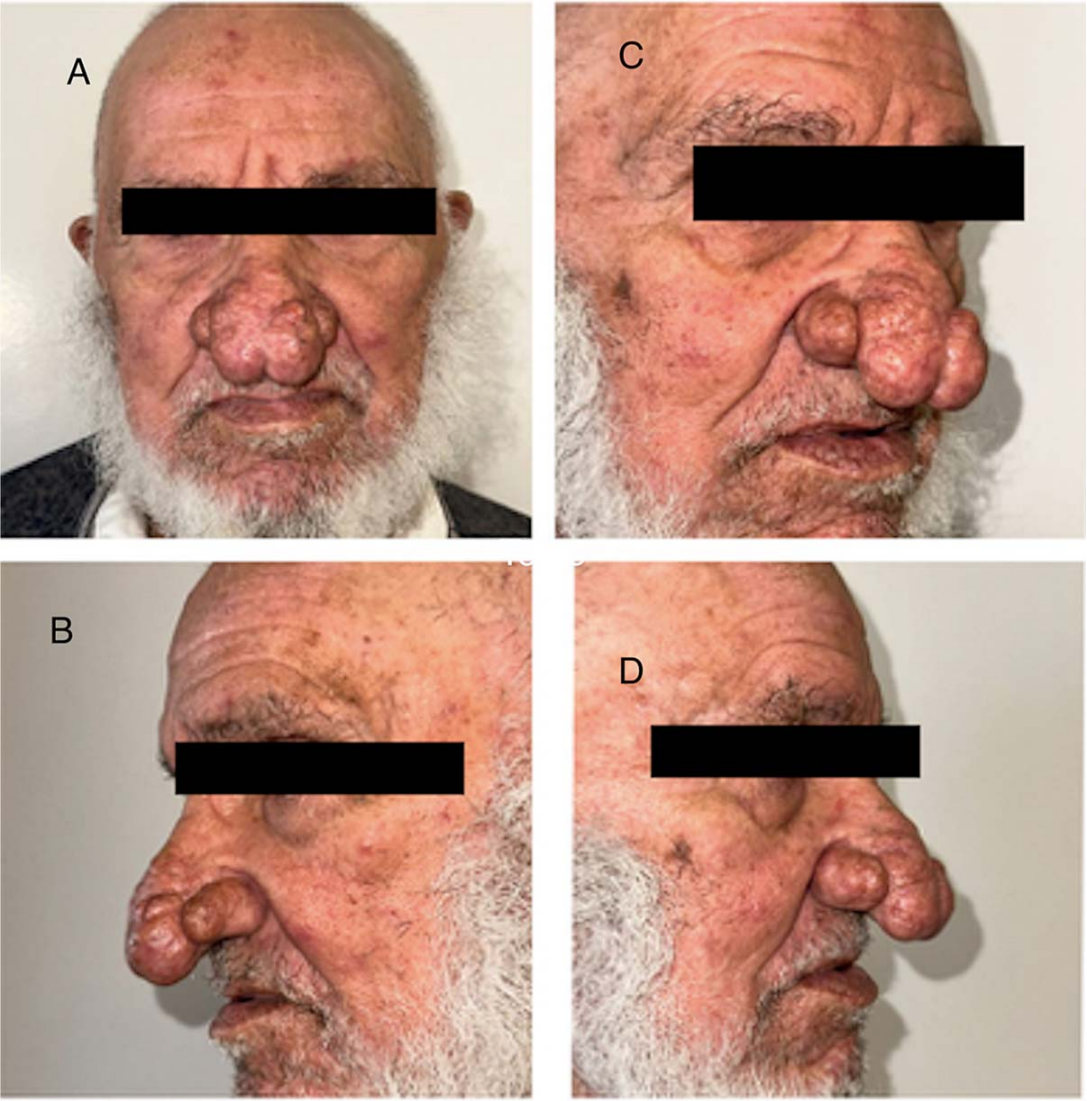
Patient 2. (A) Front view. (B) Left profile. (C, D) Right profile.

We report the cases of two patients, a 62-year-old male and an 80-year-old male, who presented with progressive nasal enlargement, redness, and acne-like lesions following a 5–7-year history of worsening rosacea, both patients presented to our department with complaints of increasing nasal enlargement, irregular skin texture, and noticeable red nodules. He reported a slow progression over several years, over a span of 4 years for the first patient and 2 years for the second, with their noses becoming progressively more bulbous and swollen (Figs 1 and 2).

**Figure 3:**
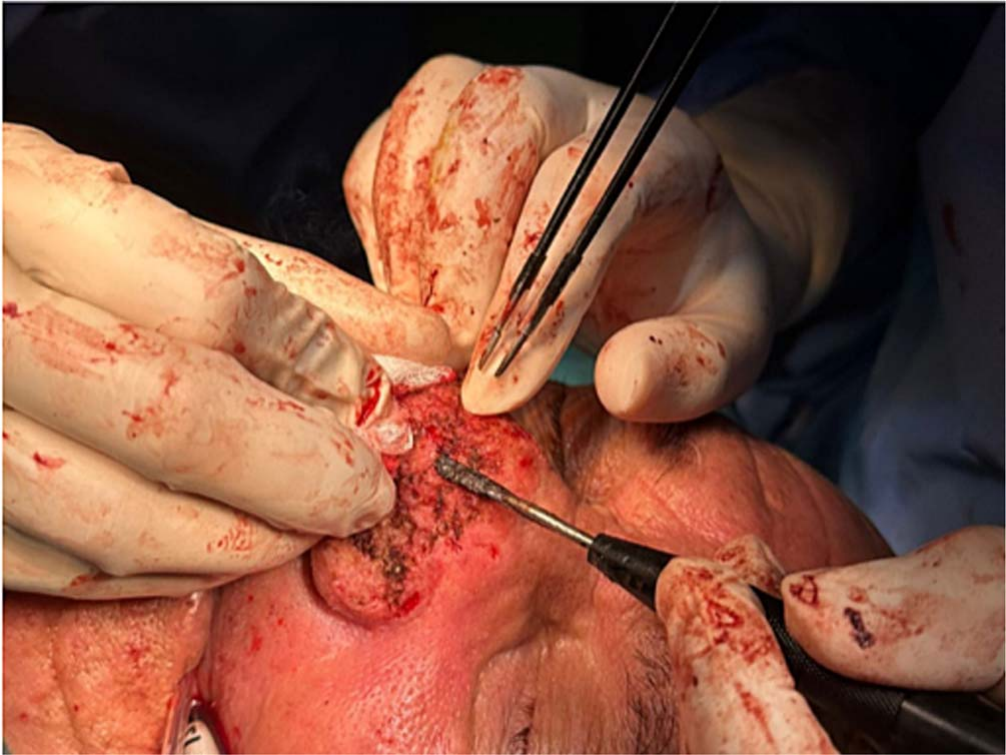
Electrocautory to reduce the mass and carving the surface of the nose using the blade tip.

Neither of the patients exoerienced any pain, but the symptoms affected their self-esteem and social interactions. Diagnostic tests included clinical examination. Treatment consisted of surgical excision of excess nasal tissue and electrosurgery (Fig. 3) under general anesthesia for both patients.

**Figure 4:**
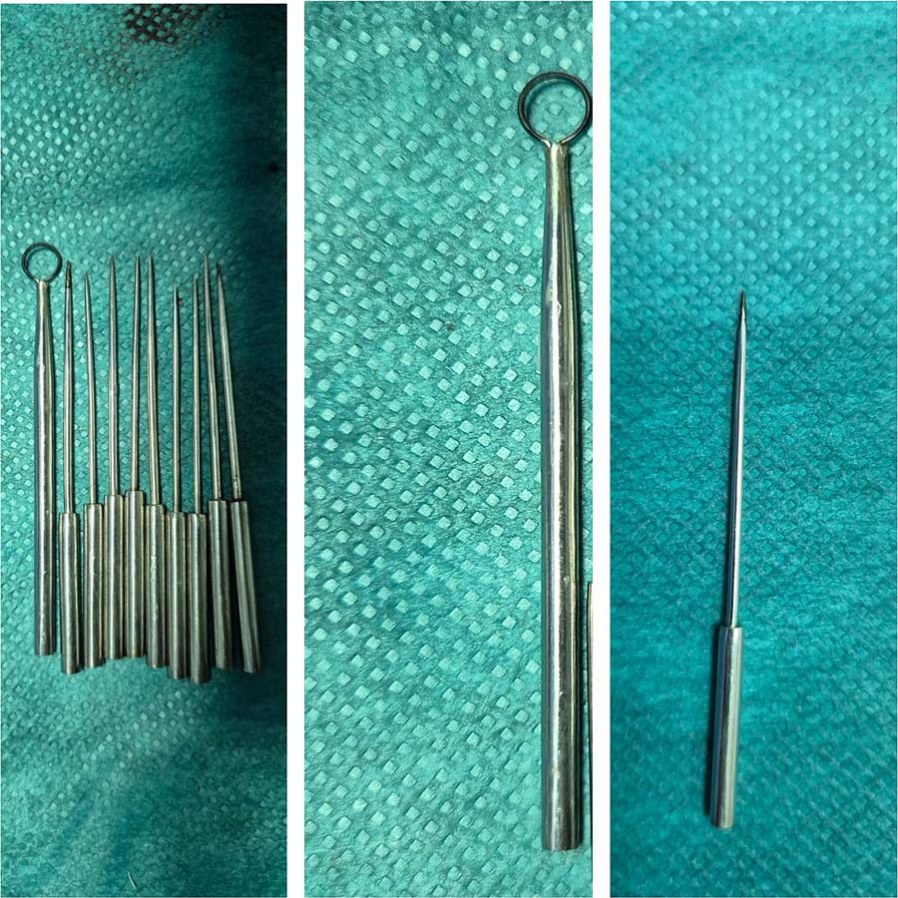
Different type of electrosurgery pencil’s tip used; in the middle the loop tip and on the left the needle tip.

This technique involves the application of a high-frequency electrical current to the tissue to achieve various effects, such as cutting, coagulation, and vaporization of tissue. Electrosurgery can be particularly effective in the treatment of rhinophyma as it allows for precise removal of thickened or fibrotic tissue while minimizing bleeding as well as reshaping the nose. In both cases the electrosurgery pencil was used with a loop tip and blade tip in addition to the needle tip (Fig. 4) following the three primary modes of electrosurgery: the cutting current, the coagulation current and desiccation mode.

**Figure 5:**
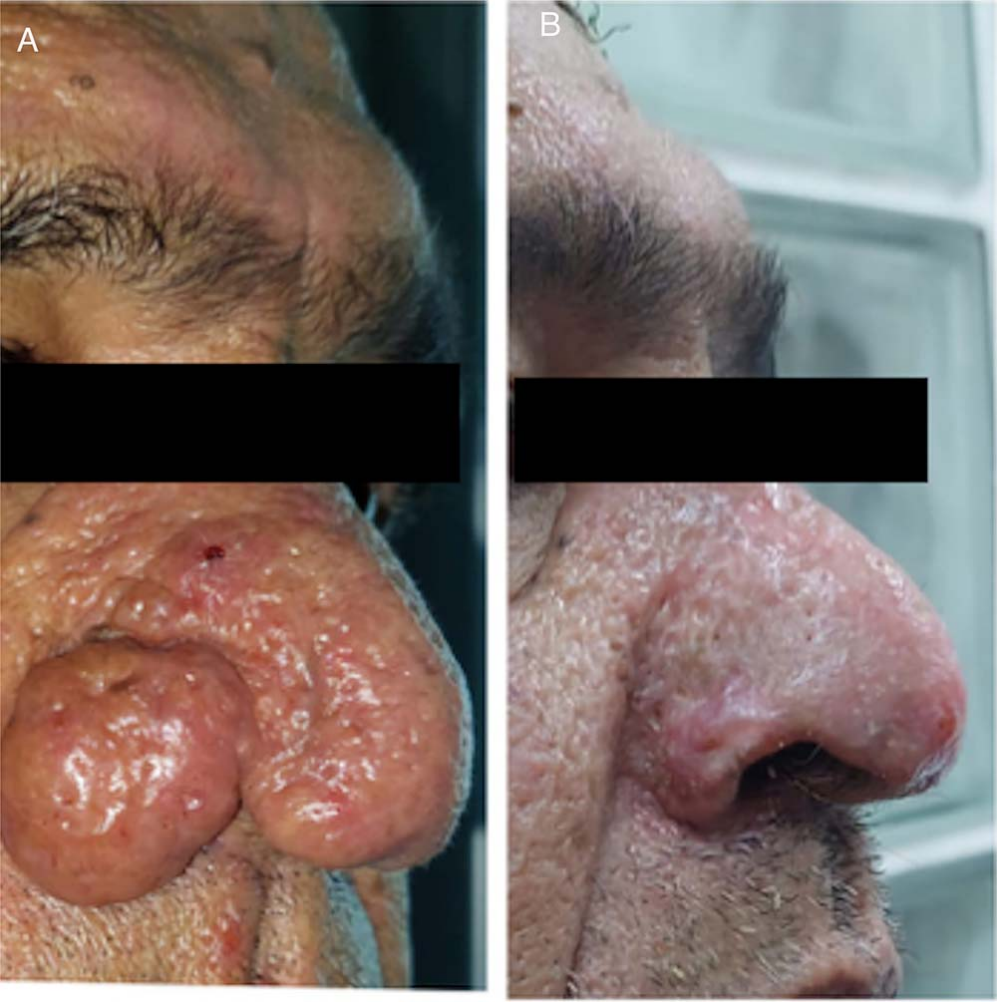
Patient 1. (A) Before the surgery. (B) After the surgery.

**Figure 6:**
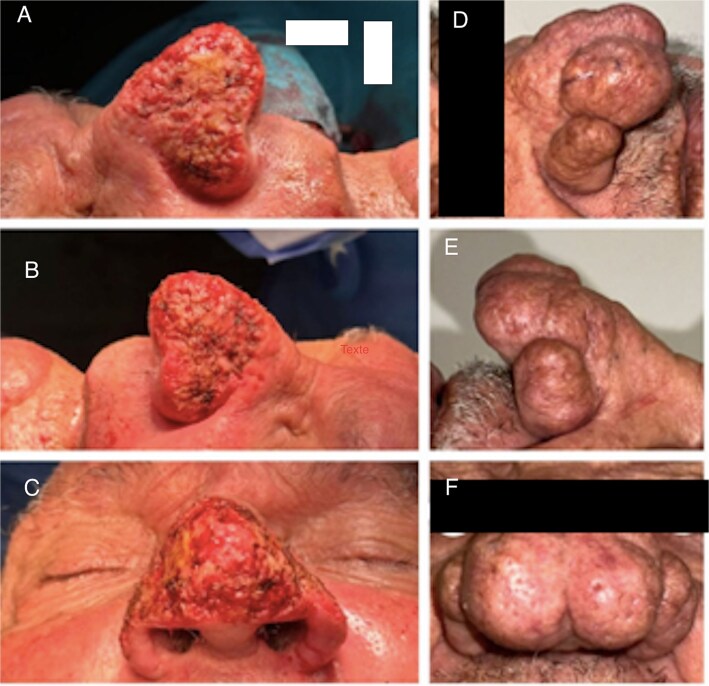
Patient 2. (A–C) After surgery. (D–F) Patient before surgery.

Both cases showed significant improvement, with Patient 1 (Fig. 5) exhibiting enhanced nasal appearance and reduced symptoms, and Patient 2 experiencing substantial symptom reduction and improved quality of life (Fig. 6).

## Discussion

Rhinophyma is the most advanced form of rosacea, a dermo epidermal disease of the face. It has long been confused with acne, and the term ‘acne rosacea’ should be abandoned [1]. The frequency of rosacea is estimated at between 0.5 and 10%, depending on the populations studied. It most often affects northern European subjects of Celtic type (fair skin, eyes and hair) [2]. It predominates in women (sex ratio 2:1) [3], peaking between the ages of 40 and 50 [4]. It usually evolves in stages, but passage from one to another is not obligatory. There are four stages: flushing; erythemato telangiectatic rosacea; rosacea with papule pustules; skin thickening or phyma.

The phyma stage is seen mainly in men (sex ratio 12/1) after the age of 50 years [5]. The exact frequency of phyma remains unknown. Familial cases have been reported in England and Ireland, but genetic transmission has not been proven [6]. Nasal localization is the most common, but localizations in the zygomas (zygophyma) and ear lobes (otophyma) have also been reported [6]. The physiopathology of rosacea remains uncertain. The origin is most likely a primitive anomaly in the vascularization of the face, localized to the dermis, resulting in flushing, permanent erythema and vascular dilatation visible through the epidermis (rose cut). The result is permanent edema, favoring increased colonization by Demodex follicularum, a mite usually found in facial hair follicles.

This colonization would in turn cause chronic inflammation of the dermis, through the activation of TGF Beta1 and 2 cytokines, leading to progressive fibrosis of the dermis and sebaceous glands [7], progressively obstructed by a plasma cell infiltrate, thus causing their dilation and sebum retention [7]. The involvement of *Helicobacter pylori* has also been suggested [2], but was ultimately invalidated by randomized studies with control subjects. Contrary to popular belief, alcoholism is no longer recognized as an etiopathogenic factor in the occurrence of phyma [7]. There is no consensus on the treatment of rosacea at the phyma stage. However, two essential elements are important to know medical treatments alone are ineffective; nodular forms are exclusively surgically treated. Surgery should be performed under the cover of probabilistic anti staphylococcal antibiotic therapy administered systemically and locally to limit the risk of postoperative chondritis [6] this treatment can be adapted if samples are taken intraoperatively and depending on the results of bacteriological cultures. On the other hand, in literature, there is no consensus or recommendation regarding the duration of treatment. Possible treatments are [6]: laser (CO2, Argon, Nd: YAG); mechanical dermabrasion: hydro dissection (Versa jet system); surgical excision.

The diagnosis of rhinophyma is clinical. The main diagnostic elements are a dyschromic appearance of the skin which is red-purple, thickened, with dilated pores, a significant amount of sebum, an irregular, bumpy and nodular surface in the most advanced forms [5]. Chondritis of the nasal cartilages may occur [5]. For the most severe forms, the progression time is between 6 and 8 years [6]. At this stage, the patient’s complaints are aesthetic, but also functional: nasal obstruction, amputation of the visual field, difficulty eating due to ptosis of the mass in front of the oral cavity, as in the case presented. It may be associated with lymphedema, particularly of the suborbital region, and, more exceptionally, ophthalmological involvement such as blepharitis, conjunctivitis and keratitis [2, 6]. A consultation with an ophthalmologist should therefore be requested for ocular signs. Rhinophyma can often be confused with other conditions that cause similar nasal deformities or tissue changes such as lupus erythematosus, sebaceous hyperplasia, cutaneous sarcoidosis, acne vulgaris and basal cell carcinoma (BCC). It is therefore strongly recommended to do a biopsy under local anesthesia, in case of doubt and depending on the context.

Rhinophyma can be treated using various methods, such as medical [7] approaches like systemic isotretinoin and doxycycline, dermal shaving, electrosurgery, carbon dioxide laser therapy with re-epithelialization [2], and surgical excision each method has distinct advantages and disadvantages, making careful selection crucial depending on the patient’s specific presentation [3].

Our case strongly advocates surgical excision of excess nasal tissue and electrosurgery as well as loop electrocautery dermabrasion to be the mainstay of treatment as it allows for the best control of hemostasis in the operating room and final cosmetic result. Electrocautery dermabrasion is also less invasive compared to surgical excision and only requires one trip to the operating room, but using an incorrect technique or prolonged application of heat can lead to thermal injury to surrounding tissues, causing burns, necrosis, or excessive scarring.

Cold scalpel excision is a cost-effective and efficient method, providing more precise tissue removal, better preservation of pilosebaceous units, and quicker re-epithelialization. However, it does have some limitations, such as inadequate hemostasis during the procedure and limited visibility of the surgical site, which often requires electrocautery [3]. In our cases, we chose to combine both techniques to improve outcomes. The Shaw scalpel offers similar benefits to cold excision, but with the added advantage of integrated hemostasis. However, it comes with an increased risk of thermal injury and potential complications.

Rhinophyma can often be confused with other conditions that cause similar nasal deformities or tissue changes such as lupus erythematosus [4], sebaceous hyperplasia, cutaneous sarcoidosis, acne vulgaris and BCC.

## Conclusions

Currently, surgical excision and/or electrocautery with dermabrasion are considered the most effective treatments for large rhinophymas. We recommend electrocautery dermabrasion as the primary approach, as it provides smooth contouring, effective hemostasis, better control during the procedure, and eliminates the need for multiple sessions.
